# A Personalized Medicine Approach is Best for Patients with Homozygous Familial Hypercholesterolemia

**DOI:** 10.18103/mra.v12i12.6160

**Published:** 2024-12-24

**Authors:** Ana Morales, Allison Jamison, Robert A. Hegele, Linda Hemphill, Samuel S. Gidding

**Affiliations:** 1 Department of Genomic Health, Geisinger, Danville, PA, USA; 2 Family Heart Foundation, Pasadena, CA, USA; 3 Schulich School of Medicine and Dentistry, Western University, London, Ontario, CA; 4 Corrigan Minehan Heart Center, Massachusetts General Hospital, Boston, MA. USA

## Abstract

Homozygous familial hypercholesterolemia (HoFH) is an autosomal semi-dominant condition characterized by biallelic pathogenic variants impacting low-density lipoprotein receptor (LDLR) function. Affected individuals have severely elevated LDL cholesterol, early onset atherosclerotic heart disease and/or aortic stenosis, and characteristic clinical findings. While the cause is known and diagnosis is relatively simple, real-world HoFH care presents many complexities, including genetic heterogeneity and the diverse personal and social circumstances that influence care. Genetics-informed treatment involves a trial-and-error approach that warrants specific considerations during pregnancy. Thus, HoFH care requires a deep understanding of personal factors, social determinants of health, and a flexible, adaptable approach to treatment, all of which justify the need for personalized care. Framed by complexity theory, this review offers strategies for personalizing HoFH care, including a reconceptualization of the definition of health and implementing a multidisciplinary team approach. We also recommend integrating complexity theory and systems thinking into clinical care. By doing so, we illustrate the advantages of classifying knowledge complexity to inform clinical decision-making. We also demonstrate how openness to relationship-building and time investment is critical to materializing personalized care to HoFH.

## Introduction

Homozygous familial hypercholesterolemia (HoFH) is characterized by the presence of two alleles limiting low density lipoprotein receptor (LDLR) function, thus causing severe elevations in LDL cholesterol (LDL-C) and consequent early onset atherosclerotic heart disease and/or aortic stenosis in affected individuals.^1,2^ This condition affects about 1/300,000 individuals worldwide, and is more common in areas where there are founder alleles (e.g., South Africa, Lebanon, Quebec, the Amish) and/or higher rates of consanguinity. The goal of treatment is to prevent cardiovascular complications by lowering LDL-C. Treatment begins at diagnosis and is both complex and intense; multiple medications and/or lipoprotein apheresis are required to lower LDL-C to safe levels and monitoring of cardiovascular status to identify severe atherosclerosis and aortic stenosis is necessary because of the potential for rapid disease progression. Late diagnosis occurs too often, well after chronic exposure to elevated LDL-C has led to premature development of atherosclerosis.

Superficially, as a medical problem, HoFH appears straightforward. The cause is known: i.e., presence of biallelic pathogenic variants in one of four causal genes (described below).^1^ Clinical characteristics are easily recognized: extreme elevation of LDL-C (typically above 400 mg/dL or 10 mmol/L), often with physical findings of xanthelasmas, xanthomas, corneal arcus, arterial bruits, or a heart murmur, and early onset of heart disease.

In clinical practice, however, HoFH care presents many hurdles, with some being insurmountable. At the provider level, the biggest challenge is identifying effective treatments. The genetic heterogeneity of HoFH impacts medication efficacy; each person responds differently to lipid lowering medications and the most effective regimen must be found via a trial and error process.^1,2^ Most individuals with HoFH will not achieve treatment goals with conventional therapy. Access to lipoprotein apheresis, clinical trials of new treatments, and sometimes, liver transplantation is needed. The lipid specialist must integrate all aspects of HoFH care: ensuring accuracy of diagnosis and interpretation of genetic test results, making decisions about treatment, coordinating multi-specialty consultation, and aiding the person with HoFH in navigating the healthcare system.

People with HoFH face high costs, severe time demands necessitated by treatment, barriers to access to care, time delays to accurate diagnosis and referral to experienced specialists, and either the fear of experiencing a cardiac event or living with the consequences of one. Most importantly, any family that includes someone with HoFH has multiple members with heterozygous FH (HeFH), perhaps others with HoFH, and confronts the issue of future family members being affected if they inherit the condition. At the healthcare system level, resources to support the complex model required for HoFH care, including experienced clinicians across multiple disciplines, availability of medications and lipoprotein apheresis, and family support must be organized and provided for.^3^

The above challenges summarize the need for a personalized medicine approach to HoFH care.^3^ At diagnosis, an understanding of *LDLR* function and genetics is required to plan a course of treatment. Each affected person and family will present with a unique set of resources, barriers to optimal care, and social circumstances. Every health care system will have both facilitators, such as existing specialists and programs, and barriers, such as lack of access to certain medications or apheresis or specialists, to care delivery.

The purpose of this paper is to present a comprehensive overview of HoFH care from the perspective of a personalized medicine approach. We will begin with a discussion of complexity theory and how it might apply to personalized medicine and HoFH. This will be followed by sections on the genetics of HoFH and its impact on diagnosis, treatment, and counseling. We integrate the personal factors impacting FH care and social determinants of health, describing how these elements make each person with HoFH unique and how these elements guide care. We will then integrate this information into a strategy for providers to personalize HoFH care. Finally, we will return to the complexity model to provide a summary and recommendations.

## Complexity Theory and Personalized Medicine

Personalized medicine requires a shift to a paradigm that allows for incorporating diverse factors from disparate sources into treatment plans. Complexity theory provides such a paradigm.^4^ Caring for HoFH can be conceptualized as a complex adaptive system (CAS), in which knowledge constantly changes, and local, often unpredictable operating rules are created by the people involved in the decisions that affect them (individuals with HoFH, families, and care providers).^5,6^ The HoFH CAS is defined by the multiple genes, variant types, and inheritance patterns associated with HoFH impacting medication response^3,7^ and the diverse personal and social circumstances in which care must be provided to promote health. These factors are summarized in the somato-psycho-socio-semiotic (SPSS) model, which posits that each individual derives their definition of health based on a balance of somatic (e.g., LDL-C levels, prior cardiac events), psychological (e.g., empowerment), semiotic (e.g., interpretation of experience), and social factors (e.g., family support).^8^

Clinicians can apply the SPSS model to generate knowledge and perspectives by exploring values and preferences across somatic, psychological, semiotic, and social domains. It may turn out that, for example, one person with HoFH may have well-controlled LDL-C but struggles with family relationships and does not consider themselves healthy, while another feels spiritually empowered and in good health despite struggling with reaching LDL-C goals. As such, the SPSS model embraces complexity by viewing health beyond disease absence, focusing on improving the overall human experience.^9^

While HoFH is a complex condition, the personal, medical, and social data in each case can be classified into simple, complicated, complex, or chaotic domains.^10–12^ Simple knowledge involves known linear facts. Operating in the simple domain represents best practice. It dictates observing or sensing first, then categorizing following known patterns and responding based on established protocols. The complicated domain includes situations that are less obvious than those in the simple domain, but linear cause and effect can still be identified with expert analysis. Within the complicated domain the aim is good practice, acknowledging that facts and context may change, requiring a new analytical approach. Complex contexts are unpredictable, have incomplete data, and lead to experimental and emergent practice. The recommended sequence of action requires acting first, determining if the action worked, and then adjusting the approach based on results. In contrast to the complex domain, where cause and effect may be retrospectively identified, the chaotic domain represents novel practice without any cause and effect. Chaotic situations demand crisis management, thus requiring direct action to control escalation, followed by sensing whether the intervention worked, and lastly, gathering the experience to generate a response, such as documenting the situation and outcomes.^10–12^

## Correct Diagnosis Improves Personalization of Care

### Diagnosis: phenotype versus genotype.

Phenotypically, plasma LDL-C is central to diagnosing HoFH.^1^ The 2023 updated consensus statement from the European Atherosclerosis Society (EAS) indicates that untreated LDL-C levels exceeding 10 mmol/L (~ 400 mg/dL) strongly suggest HoFH.^1,13^ Further evaluation is necessary to confirm the diagnosis, which should include a comprehensive medical and family history, and potentially genetic testing. Additional signs that may indicate HoFH are the presence of cutaneous or tendon xanthomas before the age of 10 and/or untreated elevated LDL-C levels consistent with HeFH in both parents.^1,13^

When any individual’s LDL-C levels are elevated, healthcare professionals should rule out secondary non-genetic causes. These include hypothyroidism, nephrotic syndrome, obstructive liver disease (such as primary biliary cirrhosis), anorexia nervosa, and such medications as corticosteroids, diuretics, non-cardioselective beta-blockers, and anti-retroviral drugs.^14^ Severely elevated LDL-C in the absence of these secondary factors suggests an underlying genetic cause.

### Genetic architecture.

HoFH is an autosomal semi-dominant disorder, meaning that individuals with one pathogenic DNA variant (i.e. HeFH) display an abnormal biochemical phenotype, sometimes accompanied by clinical findings (Table 1).

Those with two pathogenic variants, usually inherited from both parents, i.e., genotypic HoFH, experience significantly higher LDL-C levels and more severe clinical manifestations from a young age.^1,13^

To confirm a genetic diagnosis of HoFH, biallelic pathogenic or likely pathogenic variants must be identified.^1^ Most pathogenic variants are found in three main genes: *LDLR* encoding the LDL receptor (85–90% of all causal variants), *APOB* encoding apolipoprotein (apo) B (5–10%), and *PCSK9* encoding proprotein convertase subtilisin/kexin 9 (1–3%).^1,13^ A rare autosomal recessive form known as autosomal recessive hypercholesterolemia (ARH), which accounts for <1% of cases, is caused by biallelic loss-of-function variants in *LDLRAP1* encoding LDL receptor adaptor protein 1, with heterozygotes having normal LDL-C levels.^1,13^

### Allelic heterogeneity.

Most people with phenotypic HoFH have two distinct pathogenic variants (biallelic), which can either be in the same (monogenic) or different (digenic) genes. In the past, nomenclature for HoFH included confusing and complex terms like “compound heterozygous” or “double heterozygous” HoFH. To clarify this complexity, the 2023 EAS consensus statement suggested using the term “identical variant, same gene” rather than “simple homozygote,” “different variants, same gene” rather than “compound heterozygote,” and “digenic” or “different variants, different genes” rather than “double heterozygote.”^1^ Also, the statement recommended that the term “phenotypic HoFH” be used as a practical clinical shorthand recognizing that a genetic diagnosis is not available or if the results are not consistent with the clinical picture.^1^

Pathogenic variants in the *LDLR* gene are typically categorized as “null” (with <2% activity) or “receptor defective” (with 2–70% activity) based on their experimentally observed or predicted effects on receptor function. However, ≤10% of these variants have actually undergone direct functional testing; most have been classified using predictive algorithms, especially missense variants.^1^ Pathogenic variants in the *APOB* gene occur within sequences encoding the receptor binding domain while pathogenic variants in *PCSK9* occur throughout the gene but are associated with a gain-of-function of the gene product.^1^ Further illustrating complexity, there are rough but not perfect correlations between genotype and phenotype. For instance, individuals with *LDLR* null variants show the most severe LDL-C levels and the least responsiveness to drug treatments, although there is considerable inter-individual variation and genotype alone cannot definitively predict the exact clinical response.^1,13^

### Differential genetic diagnosis.

In addition to ruling out secondary non-genetic causes, clinicians should also consider other genetic conditions that can lead to elevated LDL-C levels before settling on a diagnosis of HoFH. These conditions include sitosterolemia (or “phytosterolemia”), which results from biallelic rare pathogenic variants in the ATP-binding cassette transporter genes *ABCG5* and/or *ABCG8*.^1^ Sitosterolemia is characterized by elevated LDL-C levels that respond well to dietary changes, ezetimibe, and/or bile acid sequestrants.^1^ Another condition is lysosomal acid lipase (LAL) deficiency, caused by biallelic pathogenic variants in the *LIPA* gene; enzyme replacement treatment is available, namely sebelipase. People with cerebrotendinous xanthomatosis may exhibit xanthomas similar to those seen in HoFH, but their plasma cholesterol levels are normal to mildly elevated, with elevated cholestanol levels instead, along with neurological, cognitive, and ophthalmic symptoms.^18^

## Impact of Genetics in HoFH Clinical Practice

### Benefits of genetic testing.

Overall, expert review panels have determined that the advantages of genetic testing in HoFH outweigh its limitations.^1,19^ The benefits include: 1) providing a definitive diagnosis; 2) facilitating access to appropriate current and emerging therapies and clinical trials; 3) potentially predicting disease severity and treatment responses within specific populations; 4) ruling out similar conditions that might require different treatments; 5) prompting cascade screening to identify additional at-risk relatives; 6) informing prenatal genetic counseling; and 7) possibly promoting adherence to optimal therapies.^1,19^ For example, genetic testing has identified causal variants in genes that typically cause non-FH dyslipidemias, like *ABCG5* or *ABCG8* in sitosterolemia, which has specific treatment implications such as eliminating dietary sterols and opting for ezetimibe instead of statins or apheresis.^1,19^

### Limitations of genetic testing.

Limitations of genetic testing for HoFH include: 1) issues related to accessibility and cost; 2) inconsistent predictions regarding an individual’s phenotypic characteristics and medication responses; 3) difficulties in determining the pathogenicity of many identified DNA variants; 4) discrepancies between genotype and phenotype; and 5) a lack of professional genetic counseling available both before and after testing. Healthcare providers need to exercise caution in interpreting genetic results, as they can be misunderstood or miscommunicated.^1,19^

Some individuals with phenotypic HoFH may have only one identified pathogenic variant due to technical limitations or undiscovered causal genes or variant types.^1,19^ Additionally, individuals with phenotypic HeFH who respond well to dietary changes and statin therapy may still possess two pathogenic variants that would typically be expected to result in phenotypic HoFH. In cases of discordance between genotype and phenotype, the 2023 EAS position statement advises prioritizing the phenotypic diagnosis over genetic findings.^1,19^ People with HoFH and families facing complex or confusing genetic results should be referred to a genetic counselor or clinician experienced in human genetics and lipidology.^1,19^

## Management and Treatment

### Predicting drug response based on genotype.

It has traditionally been believed that the effectiveness of drug treatment is linked to the level of residual LDL receptor activity associated with a pathogenic variant, although this relationship is not always consistent.^1^ For instance, some people with HoFH respond moderately well to PCSK9 inhibitor therapy, but this response cannot be reliably predicted based on genotype.^20^ If a trial of PCSK9 inhibitor therapy results in >15% reduction in LDL-C over and above existing therapies, treatment may continue.^20^ However, if the response falls below this threshold, clinicians should consider discontinuing the therapy and expeditiously explore more predictable and effective options. For example, LDL-C responses to evinacumab,^21^ lomitapide,^22^ or apheresis do not depend on *LDLR* genotype and tend to be similar in individuals with biallelic null variants or those with predicted residual LDL receptor function.

### Pregnancy.

Women with HoFH should receive coordinated care from a multidisciplinary team, which includes cardiovascular assessments before, during, and after pregnancy. To reduce LDL-C exposure, apheresis should be offered during pregnancy, and statin therapy along with other lipid-lowering treatments should be resumed starting in the second trimester.^1^

### Lipoprotein apheresis.

Lipoprotein apheresis as currently implemented in the United States mechanically separates plasma from red blood cells to remove circulating LDL particles through adsorption to dextran sulfate coated beads and then returns the plasma and red blood cells to the circulation; other similar systems are available worldwide.^23^ Lipoprotein apheresis has several advantages as a therapy for HoFH. It works independently of LDL receptor function. In homozygotes it lowers LDL-C by a time averaged 35% and 46% if performed bi-weekly and weekly, respectively.^23^ In addition, apheresis has pleiotropic benefits.^1,2,24^ Lp(a) levels are also lowered substantially, and vascular inflammation, critical for atherosclerosis progression, is substantially improved.^25,26^ While no clinical trials of lipoprotein apheresis have been performed, substantial observational data suggest that it is a highly effective therapy, including natural history studies showing improved outcomes in those receiving apheresis and reduced event rates in high-risk individuals after starting the procedure compared to baseline.^27,28^ Apheresis remains among the few approved treatments that can be used during pregnancy. While treatment frequency can be lowered in the context of effective use of medication, many individuals still cannot achieve treatment goals.^1,2^

However, lipoprotein apheresis typically remains among the last treatments offered, because of limited access. Apheresis requires weekly or bi-weekly 2–3-hour sessions at a specialized apheresis center. Many countries lack apheresis capacity. Furthermore, even if a center is available, transportation may be challenging for many people with HoFH and their families. Often a venous port needs to be inserted or a fistula created surgically to provide serial vascular access. Medical complications include milder symptoms, including lightheadedness, nausea, and numbness/tingling, while more serious complications include hypotension, headache, anemia, chest pain, allergic reactions, and abdominal discomfort. Side effects rates are relatively low, with a frequency of ~5%.

The need for lipoprotein apheresis adds substantial complexity to care as it has a significant impact on the lifestyles of both affected and unaffected family members due to logistical issues related to transportation, time lost from work and other activities, and financial considerations if out-of-pocket expenses are not covered by health insurance.^29^ Several studies of quality of life in people with HoFH undergoing apheresis have somewhat conflicting results.^29,30^ Physical, emotional, psychological, and aesthetic problems are common, including fears related to long term prognosis. Younger people with HoFH appeared to cope better.

## Personal and Social Considerations Influencing HoFH Treatment

Individuals living with HoFH are fortunate to have many more treatment options available now than even just ten years ago. Given the severity of health impacts, it is imperative that providers pursue combined lipid lowering treatments to maximize effectiveness. However, in determining the best combination of therapies, the provider must also consider which are most appropriate and likely to be consistently tolerated considering the individual’s age, gender, financial resources, and cultural and religious beliefs.

### Age.

Recent research indicates that only the most severe presentations of HoFH are diagnosed in childhood, so many individuals living with HoFH may be older at time of diagnosis.^31^ Providers must balance the limitations and challenges of each treatment option and its impact on an individual at different life stages.

With children, many first-line therapies are oral medications - statins, ezetimibe, bempedoic acid - that may be difficult for a child to swallow. This may in turn make it difficult to ensure compliance. Bile acid sequestrants, while available in suspension form, have a bad taste and can lead to gastrointestinal side effects. Newer treatments, including inhibitors of PCSK9, and angiopoietin like 3 protein (ANGPTL3) involve infusions or injections, which younger individuals may find frightening or even traumatizing. Parents may therefore be reluctant to adhere to regular injections for children.

Similarly, LDL apheresis involves needles as well as likely minor surgery for port implantation for access. Injections and infusions may also require administration of the drugs in a hospital setting, which requires children to regularly miss school. Apheresis often requires hospital administration as well, and frequently also requires a child and a caregiver to travel, sometimes long distances, to an apheresis center. Liver transplantation, which can be curative for HoFH, involves major surgery and lifelong monitoring and medication to prevent rejection of the donor liver.

In all treatment contexts, there is also the realization that a child who was previously believed to be healthy is now considered “sick,” and parents and/or children may fear the stigma of being a medically fragile child. Children may resist treatment because they do not feel sick. Parents’ worries result in children being withheld from sports or other activities, and dietary concerns can lead to missing out on special events, or in some cases even disordered eating. Providers and parents need to consider age-appropriate communication for children living with HoFH to explain the need for care without raising alarm.

Adults with HoFH may have similar concerns. LDL apheresis, in particular, may be more challenging for adults with respect to expenses, travel, and time commitments. In the US, most adults depend upon employment for health insurance, leading some individuals with HoFH to hesitate when requesting required time off for apheresis. Adults also have other obligations – to family, children, employers, and others – that may be in conflict with the time required for apheresis.

Individuals who are diagnosed later in life may also lack motivation to pursue treatment aggressively, especially if they have not yet experienced a cardiac event. Adults may fear side effects of both oral and injectable medications and can succumb to influence from social media groups that spread fear and disinformation regarding pharmaceutical options.

### Gender.

Perhaps the biggest gender difference in treatment of HoFH is pregnancy. Most oral and injectable available treatments for HoFH are contraindicated during pregnancy. However, women living with HoFH who plan to become pregnant may be able to start or continue bile acid sequestrants and/or LDL apheresis. If a woman with HoFH already has significant atherosclerotic cardiovascular disease (ASCVD), she may face additional risks during pregnancy and delivery.

For men living with HoFH, studies show that ASCVD end points occur earlier than in women. Men should therefore be made aware of their risk and of the symptoms of heart attack and stroke. Women are more likely to experience atypical ASCVD symptoms and are less likely to be suspected of having a cardiac event when symptoms occur. Both genders should be counseled on typical and atypical ASCVD symptoms.

### Financial resources.

The cost of treatment can be daunting for many people with HoFH. This includes costs for prescription drugs and LDL apheresis, but also transportation costs, time off from work or school, lost wages, and time spent working through complicated insurance requirements. While relatively inexpensive generic statins are available, newer drugs can be very expensive, depending on insurance coverage. Given financial and insurance constraints, people with HoFH may be unable to access or afford some currently available treatments. Additionally, individuals may hesitate to seek emergency medical care if they experience symptoms of a heart attack or stroke, due to concern over costs associated with an emergency room visit.

### Religion and cultural beliefs.

Even with severely elevated LDL-C levels seen in individuals with HoFH together with the evidence of early and aggressive heart disease, some individuals may choose to initiate dietary modification and non-pharmaceutical remedies first. A segment of people distrusts organized healthcare and pharmaceuticals, and providers may have to work through this distrust before initiating appropriate therapies. Despite no exchange of blood products, some individuals may misunderstand the apheresis process and object on the basis of religious beliefs that limit or prohibit the use of blood products. Also, social media has given rise to forums for the discussion and dissemination of non-mainstream medical views regarding the root cause of heart disease. In particular, some user groups discount the role of LDL-C in atherosclerotic disease, focusing instead on inflammation as the actual risk factor.

### Social determinants of health.

The social determinants of health (SDOH) are environmental factors that influence health.^32^ The SDOH are grouped in five domains: economic stability, education access and quality, healthcare access and quality, neighborhood and built environment, and social and community context.^32^ For example, the geographic location of someone with HoFH can determine access to effective therapy, which in turn can affect an individual’s LDL-C. Disease itself can have economic and social effects. For example, individuals with HoFH may experience a diminished quality of life, increased healthcare costs, and reduced work productivity. These effects may ripple out to impact communities.

Research on the quality of life for individuals living with HoFH is limited. The impact on quality of life may vary based on the individual’s disease status and treatment, especially for those undergoing apheresis, which can impose a significant burden affecting education and employment. A Dutch study found that 90% of people with HoFH receiving pharmacological treatment without apheresis reported quality of life levels that were only slightly below the national average.^33^ People with HoFH experienced transient anxiety when facing the implications of HoFH, such as the risk of developing or worsening cardiovascular disease. Family planning services provided by specialized HoFH centers were beneficial in helping individuals manage their condition.^1^ Comprehensive support for people with HoFH and their families in specialized lipid clinics is recommended.^1^

**Figure 1.**
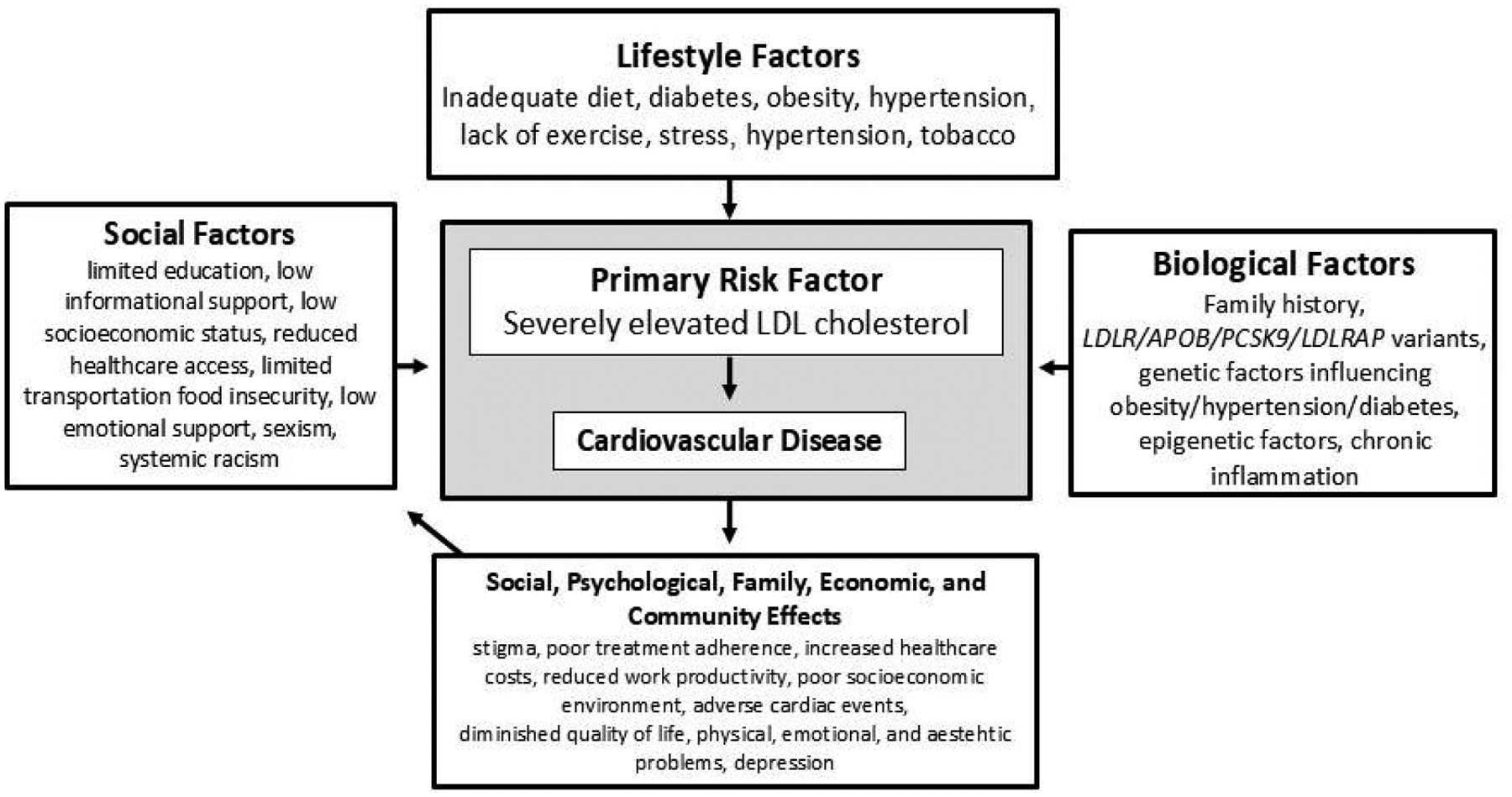
Biological, personal, and social factors influencing HoFH health. The arrows illustrate a simple interaction between these factors. Numerous complex interactions exist, but for simplicity, are not shown. Adapted from Celentano et al. 2019,^34^ Tsao et al. (2002),^35^ Powell-Wiley et al. (2022),^36^ and Brandt et al. (2023).^37^

As shown in Figure 1, severely elevated LDL-C and cardiovascular disease are influenced by lifestyle, social, and biologic factors. To illustrate one dimension of the many complex relationships involved, social, psychological, family, economic, and community effects not only result from disease but also influence social factors, creating a feedback loop. These factors serve as the building blocks of the HoFH CAS. Understanding this dynamic interplay facilitates learning, managing knowledge, and implementing personalized treatment plans or, returning to the SPSS model, a personalized definition of health for different people.

## Genetic Counseling

The primary functions of genetic counseling are to assist the person with HoFH and their family to understand diagnostic testing options and familial risk, to assist at-risk relatives in pursuing testing, and to offer psychosocial support throughout the process. Offering psychosocial support to adapt to a diagnosis and risk is paramount to the genetic counseling process. For example, given the underdiagnosis of FH, it is possible that a child with HoFH may be diagnosed at the same time as their parents are diagnosed with HeFH. Therefore, parents may have minimal understanding of the health risks the condition poses. If the family has been unaware of FH until the time of an HoFH diagnosis, they may be resistant to medication, especially for children, until they have first tried diet modification and exercise. Parents who have been living with FH themselves, without knowing it, may not have yet seen any impact on themselves and may therefore be reluctant to subject their child to HoFH treatments in general. Part of the care in these cases should include family education and connection to resources and support. The same situation may exist for parents of adopted children, as parents may have minimal or no exposure to FH or its potential impacts on health and longevity.

People receiving genetic counseling should feel as equal partners. The interactions between the person with HoFH and the clinician should be framed within complexity principles to promote autonomy and shared decision making. Three genetic counseling issues relevant to HoFH illustrate these points: 1) genetic risk assessment; 2) reproductive counseling; and 3) the decision to have a genetic test.

### Genetic risk assessment.

When a person is diagnosed with HoFH, first-degree relatives (parents, siblings, and children) are at risk for HoFH or HeFH. Most cases are inherited,^38^ that is, at least one parent is also affected. When assessing risk for children, the FH status of the partner should be established or, if unavailable, estimated based on population prevalence. While the most common pattern of inheritance of HoFH is monogenic semi-dominant and the rarest is autosomal recessive, parental genetic and lipid testing allows calculating precise familial risk.^38^ The proband’s genotype determines risk to offspring. Parental results determine risk to siblings. Rarely, parents may have negative testing. In these cases, once non-paternity and non-maternity are ruled out, the risk to siblings should incorporate the rare possibility of parental germline mosaicism.^38^

Children of an individual with semi dominant HoFH (for example, from biallelic *LDLR* variants) will inherit one FH variant and thus will have HeFH. However, if the partner has HeFH or HoFH, children will be at risk of having HoFH. On the other hand, children of individuals with digenic HoFH (for example, from biallelic variants in *LDLR* and *PCSK9*) may inherit both FH causing variants and also have digenic FH. Alternatively, they may not inherit any of the variants and be unaffected. A third scenario is that of HoFH caused by *LDLRAP1* variants, which is inherited in an autosomal recessive manner. As such, parents with one *LDLRAP1* variant are unaffected carriers.^38^ Tables 2 and 3 illustrate these and additional selected scenarios. As shown, the risk calculations can range from simple to complicated, with complexity added by rare or theoretical outcomes, such as the presence of more than two disease-causing variants, and the fact that it is impossible or impractical to predict every scenario.

### Reproductive counseling.

Both men and women should be counseled on the genetics of FH inheritance prior to pregnancy, as well as the associated risks to the mother if she is the one living with HoFH. For both genders, there is the consideration of passing along FH, either heterozygous or homozygous, to children. There can be complicated feelings of guilt and fear when considering a genetic condition, and for individuals living with HoFH in particular, in knowing that their children will likely inherit HeFH.

Reproductive counseling is best provided during the preconception period. During this stage, recommendations are more likely to be actionable and contraception can be implemented to avoid unplanned pregnancies.^1^ For a precise risk assessment, genetic testing is recommended for reproductive decision making.^39^ Testing the partner might reveal FH, which has implications for disease in future children (Table 3).

Genetic counseling risk assessment in the prenatal setting may lead to fetal testing via amniocentesis or chorionic villus sampling and consultation with a FH specialist to discuss prognosis and potential treatment if a pregnancy is affected. If a prenatal diagnosis of HoFH is made, reproductive decision-making to decide whether to continue with the pregnancy is necessary. Gamete donation, preimplantation genetic diagnosis, avoiding biological children, or adoption are alternatives to couples who have an increased probability of having a child with HoFH. Umbilical cord testing after birth is another viable option as it allows early diagnosis and treatment, if necessary. These decisions must be made within the framework of values and legal restrictions.^39^

For the individual carrying the pregnancy, the importance of therapy should be discussed in collaboration with the prescribing clinician. For example, discontinuation of lipid lowering therapy may be necessary.^39^ This conversation should include discussion about the teratogenic risks of medication. Adjustments of diet and lifestyle and apheresis in severe cases are also important. Reproductive counseling should also include the benefits from multidisciplinary care. Cardiology surveillance, in collaboration with a high-risk obstetrician, is also indicated, considering the increased hemodynamic demands resulting from pregnancy and the natural LDL-C rise during the second and third trimesters.^1^

### The decision to have a genetic test.

Given the complexities of DNA testing for HoFH, genetic counseling is desirable, although concerns have been raised about the limited availability of genetic counseling services.^1^ The condition can be diagnosed by evaluation of LDL-C levels and other clinical parameters or by DNA testing. Genetic counseling should include a discussion about the difference between DNA and LDL-C testing, outlining the benefits and limitations of both modalities.

Identifying the specific genes and variants involved informs familial and reproductive risk counseling.^1^ Compared to genetic testing, LDL-C testing is widely recognized and available, has a relatively lower cost, and is routinely ordered by clinicians. While the detection rate of genetic testing for HeFH can be up to 60%, higher detection rates^38^ may be possible for people with a clinical diagnosis of HoFH. For example, a study reported that genetic testing was positive in 40% of individuals with HeFH^40^ while all individuals with HoFH (n=41) had a genetic etiology identified. These data suggest that a genetic predisposition may be confidently inferred from a clinical diagnosis of HoFH.

The decision to have a genetic test may also be affected by concerns over genetic discrimination pertaining to disclosed genetic information. This issue is particularly concerning for individuals without a documented clinical diagnosis, resulting in health insurance or employment denial, a risk that is heightened for individuals lacking insurance at the time of testing. However, in the US, the Genetic Information Non-Discrimination Act (GINA) makes such discrimination of individuals illegal.^41,42^ This regulation complements the Americans with Disabilities Act, U.S. military, Veteran’s Health Administration, and local state laws, which also afford protections against genetic discrimination.^43^ On the other hand, protections are not in place for life, long-term, or disability insurance, or for individuals working in companies with less than 15 employees. Whether genetic discrimination has occurred is difficult to document and study. Two systematic reviews found that fear of genetic discrimination is a common and valid concern based on some documented cases.^44,45^ However, the evidence is not robust, as the number of incidents is small and may sometimes stem from technical errors rather than discrimination.^44,45^

## Personalized Medicine Care Strategy

To provide personalized care, extensive information must be acquired related to the genetic, medical, personal, and social factors described above. Some information will be acquired at the initial clinical encounters, some will be acquired with diagnostic testing, and some will emerge as new challenges arise. Table 5 summarizes this information as strategies for personalizing HoFH care across four domains: Medical, Person with HoFH and Family, Social Determinants of Health, and Care Coordination.

The medical level encompasses the traditional components of HoFH care; however, each component will need to be individualized. Each person with HoFH will have a different overall cardiovascular risk profile, absence or presence of aortic stenosis, a specific genetic variant, a variable response to medication, and a diverse family with varying degrees of interest in cascade testing. Each person with HoFH and family are unique with respect to experience with FH. Some family members with HeFH will likely have had prior heart disease and received lipid lowering treatments. Pregnancy and concerns about inheritance may create stress. Important family members will step forward and take responsibility for care coordination. Family members without FH will be equally important in family dynamics as they have many concerns, including morbidity in loved ones and financial security. Most important, understanding the individual’s concepts of health and personal safety, fears concerning morbidity, beliefs around treatment adherence, and prior experiences with the health care system will be essential for maintaining trust in the relationship with their care provider.

### The Cynefin framework as a personalized medicine care strategy.

The Cynefin framework (Welsh-inspired, pronounced kuh-neh-vin) offers a structured approach to managing complexity by facilitating the integration of medical knowledge to practice personalized medicine.^10,12,46^

**Figure 2.**
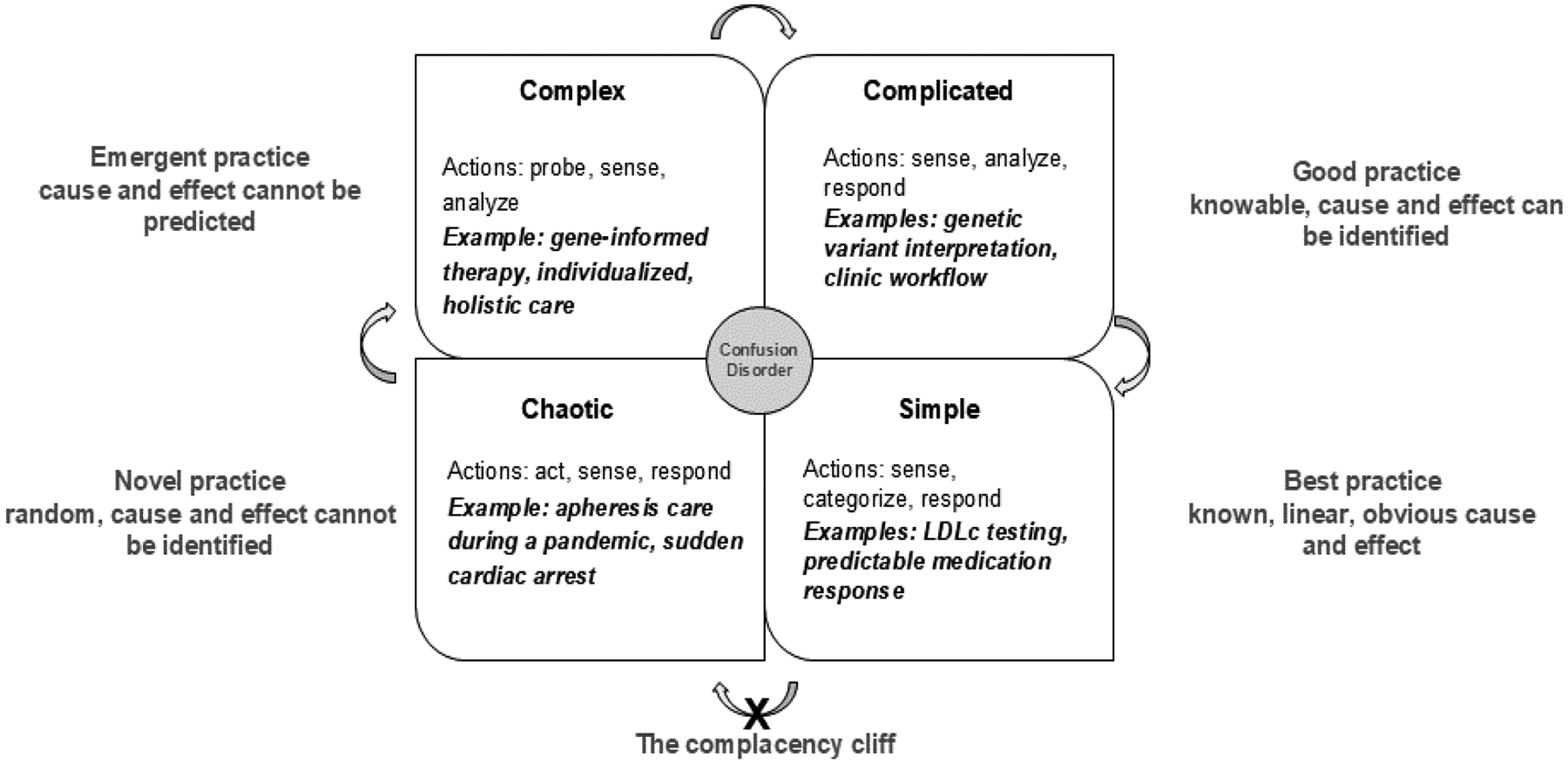
Cynefin framework applied to HoFH. Adapted from Sturmberg and Miles (2013).^11^

Figure 2 shows how the Cynefin framework can be applied to knowledge management in HoFH. Testing of LDL-C is an example of simple knowledge. Operating in the *simple* domain requires sensing first (LDL-C is 400 mg/dL), then categorizing based on standard laboratory cutoff values (LDL-C is significantly above 190 mg/dL), and finally responding based on guidelines (lipid lowering therapy and HoFH evaluation are indicated). The process of understanding the effect of an identified variant in an FH associated gene is an example of *complicated* knowledge. In this domain it is possible to identify linear cause and effect with analysis based on specialized knowledge. Through application of algorithms from variant classification guidelines,^47^ the laboratory director identifies the variant, analyzes criteria for pathogenicity, and determines that the result is positive based on the variant meeting certain criteria for pathogenicity and no contradicting evidence. Even with a pathogenic classification, variant classification is probabilistic, which illustrates the concept of good practice, considering that variant pathogenicity can be upgraded or downgraded as new evidence becomes available.^48^ An example of *complex* knowledge is genetics-informed therapy management, a trial and error process.^3^ Clinicians operating in complex contexts should use an exploratory approach to action (prescribing PCSK9 inhibitors for individuals with null or limited function *LDLR*-HoFH), then after observing the LDL-C response, determine if the intervention worked. Treatment is then adjusted based on this response. Complexity may be increased by challenges related to medication access and cost. Contrary to the complex domain, the *chaotic* domain requires action for the goal of achieving control. Chaotic situations, such as providing critical HoFH care during the COVID-19 pandemic,^49^ require acting first to prevent unleashed havoc, e.g., coordinating to ensure that apheresis service remains safe, available, and effective. Once the situation is stabilized, sensing, by gathering facts and insights about the event and actions (e.g., apheresis remained safe), follows. A response can then be formulated based on the collected information, e.g., a protocol for apheresis care during COVID-19 is developed.

As insights are gained in each domain, there is an opportunity to simplify knowledge, but simplification comes with the risk of complacency. For this reason, the boundary between simple and chaotic contexts is best described as a cliff that, if crossed, can lead to catastrophic consequences.^12^ For example, developing a plan for apheresis service during the pandemic can lead to innovative approaches and perhaps more simplified practice, but over reliance on such a tool can escape nuance in a constantly shifting context such as a worldwide pandemic.

Care coordination will be a combined task for the medical team and the person with HoFH and family. Table 6 offers our recommendations for multidisciplinary care in HoFH. Ideally a nurse or social worker can serve as a personal liaison linking the team to the family. Facilitating access to the many resources required over the life course for FH care will require anticipation of needs by the medical team and communication of perceived care gaps or need for support by people with HoFH and families. Lurking in the background will be social determinants of health, creating both barriers and facilitators to achieving successful care coordination and achievement of treatment goals.

## Recommendations

We have demonstrated that knowing LDL-C, cardiovascular state, including aortic stenosis assessment, and the causative genotype are necessary but insufficient to improve HoFH health. People with HoFH have families and live in communities with health systems of varying sophistication. We offer recommendations to move this knowledge into action.

### Practice systems thinking.

Understanding the intricate connections between medical, genetic, therapeutic, personal, and social factors is essential to providing empathetic care to people with HoFH. It’s crucial to grasp the health and illness beliefs of people with HoFH, concerns about safety, attitudes toward treatment adherence, and past experiences with the healthcare system. In addition, reproductive choices should be made within the context of personal values and legal limitations. Treatment for HoFH should be customized to meet the specific needs of the individual because of the multi-layered factors that shape a distinct picture for each person. These considerations are key to sustaining trust and illustrate how HoFH functions as a CAS.

### Identify a system’s complexity.

By applying complexity theory principles, providers can better understand the system and provide a personalized medicine approach to HoFH. Healthcare providers must integrate all aspects of care, including specialized medical expertise, accurate diagnosis with genetic testing, treatment decisions, coordination of multi-specialty care, and culturally sensitive support for people navigating the healthcare system. For instance, nomenclature issues can impact communication. This is illustrated by the 2023 EAS recommendation to use the term “phenotypic HoFH,” which can promote simplicity and finding common ground among multidisciplinary teams. A team approach enhances this integration of care with quality, including that of the person with HoFH and their family.

### Use frameworks to navigate a complex system.

Key applications that embrace complexity while integrating all aspects of care include the SPSS model to explore values and preferences across multiple domains. Applying personal factors and structured frameworks such as the SDOH enhances our ability to generate and simplify knowledge. The Cynefin framework can help foster tolerance for non-linear thinking and unknowns, which can enhance openness to novel ideas and decision-making. Importantly, the closer to chaos in the Cynefin framework, the greater the time investment required to manage and take actions based on gathered knowledge. These applications require constant monitoring to ensure proper observation, accurate classification of knowledge, and appropriate allocation of time to provide optimal care.

### Identify the simple rules that drive a complex system.

Understanding the system requires observation and sometimes acting before observation is possible. These strategies can reveal the simple rules and core values that influence behaviors, much like understanding how simple pressure differences drive a bathtub vortex.^9,54^ For example, humans are emotional beings driven to belong. Misinformation and pseudoscience about inflammation as the root cause of cardiovascular disease pushed through social media can distort someone’s perceptions of group norms to ensure belonging, potentially leading to non-adherence to therapy. It is within these simple rules, such as the desire to fit in, that barriers and facilitators to practice and compliance can be identified. In this example, an appropriate strategy for clinicians is to invest in relationship-building with people with HoFH and their family.

### Avoid oversimplification.

Knowledge simplification is desirable whenever possible; however, we view simplification as an aspirational aim. First, a CAS is continually evolving. Therefore, we should be prepared for new developments (e.g., a variant reclassified, a new drug) when we feel that all possible knowledge has been gained and clinical pathways have been standardized. Second, avoid the potential debacle resulting from complacency. Homozygous familial hypercholesterolemia is a CAS. Expecting inevitable shifts requires abandoning rigid approaches and conditioned responses in favor of adaptability and openness to continuous learning. Constant monitoring ensures proper knowledge classification and openness to novel ideas to maximize the effectiveness of medical actions. Most important, if a patient care issue is assessed as complicated, complex, or chaotic, allow sufficient time to develop an approach to the issue that can be sustained over a long period of time.

## Conclusion

Systems thinking enables appreciating complexity. Existing frameworks are available to navigate complexity in healthcare, and personalized care for people with HoFH. Oftentimes, what appears to be complex can be detangled into simpler parts. Identifying the simple rules that drive a CAS can improve strategies for care interventions. To achieve these goals, openness to relationship-building and thriving in an unpredictable complexity paradigm are key to transforming observations into meaningful actions that improve health over time.^55^

## Figures and Tables

**Table 1.** Genetic Architecture

Gene	Inheritance	Genetic mechanism	Proportion of cases or causal variants	Comments
*LDLR*	Semi dominant	Loss of function	up to 90%	Null alleles are associated with the most severe clinical presentation, while defective alleles are relatively less severe.
*APOB*	Semi dominant	Loss of function	up to 10%	HoFH phenotype is associated with defects at the receptor binding site. Biallelic truncating variants lead to a different phenotype: familial hypobetalipoproteinemia.^15,16^
*PCSK9*	Semi dominant	Gain of function	up to 3%	Loss of function variants often cause depressed LDL-C levels.^17^
*LDLRAP1*	Recessive	Loss of function	<1%	The only true recessive form, since heterozygous carrier parents have no clinical phenotype.

**Table 2.** Parental and sibling risk based on HoFH proband genotype. blue = monogenic HoFH; light blue = HeFH; white = unaffected; green = digenic HoFH; navy blue = monogenic semi-dominant HoFH in one gene and HeFH in the second gene; gray = unaffected carrier. Some rare scenarios are not shown.

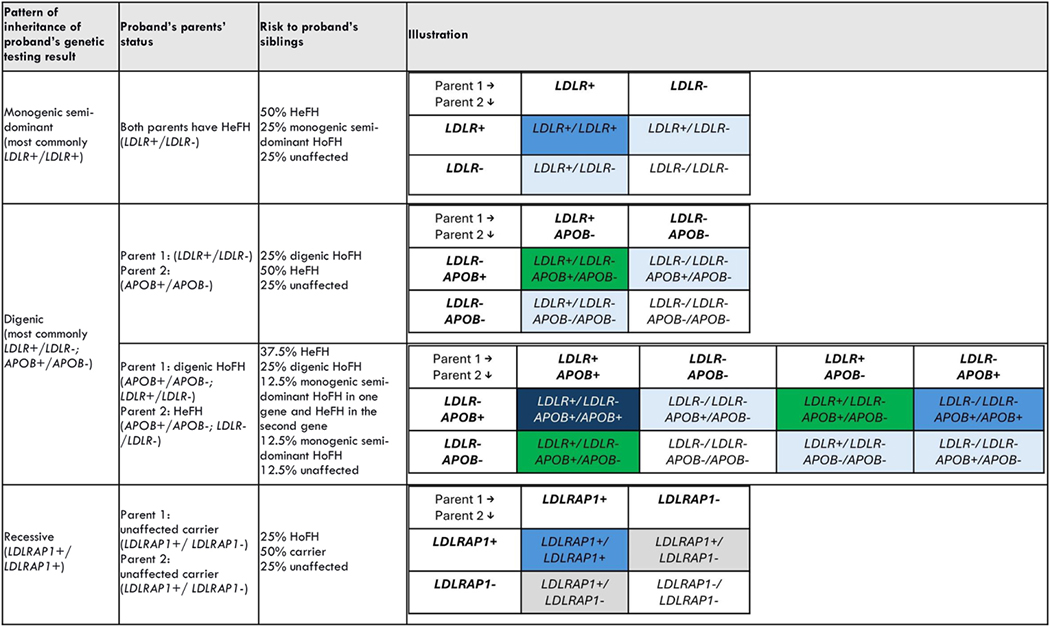

**Table 3.** Reproductive risk based on HoFH proband genotype. blue = monogenic HoFH; light blue = HeFH; white = unaffected; green = digenic HoFH; navy blue = monogenic semi-dominant HoFH in one gene and HeFH in the second gene; gray = unaffected carrier; violet = digenic biallelic HoFH. Some rare scenarios are not shown.

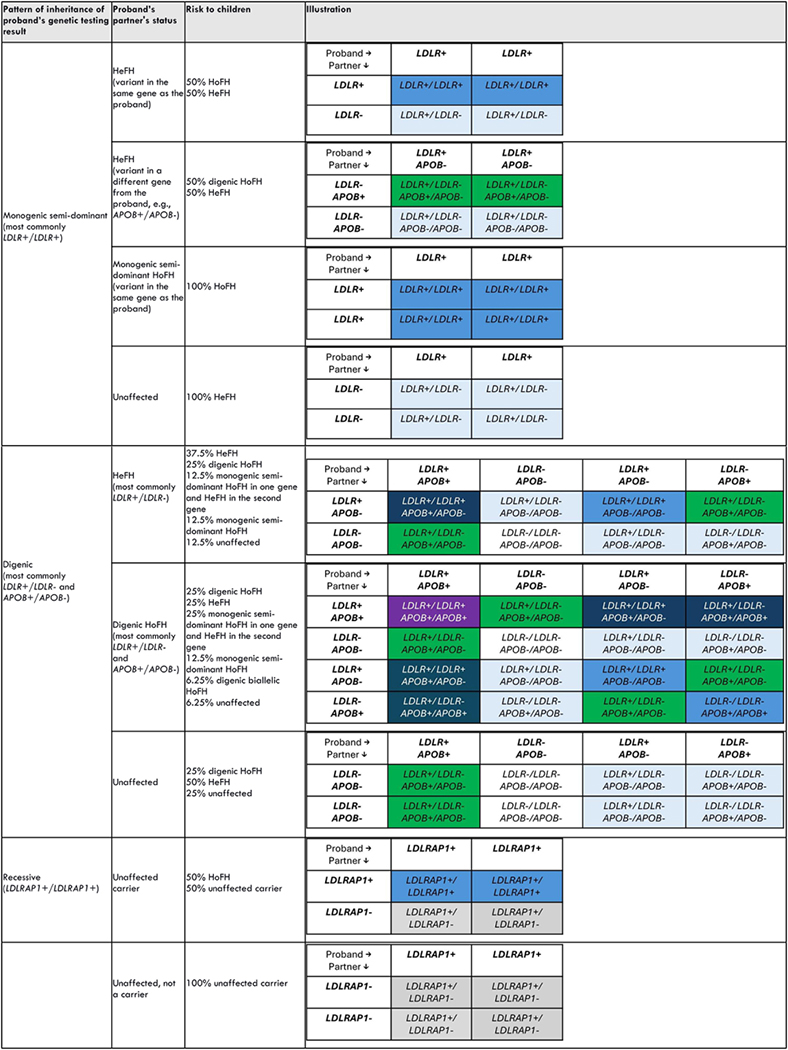

**Table 5.** Strategies for Personalizing HoFH Care

**Domain**	**Strategy**
Medical	Evaluate overall cardiovascular risk Interpret genetic and other test results Initiate cascade testing Manage treatment and disease monitoring
Person with HoFH and Family	Know the personal and family experience with FH: Heart disease Elevated cholesterol Pregnancy
	Allow time to make sure health beliefs are adequately addressed Understand concerns and fears about future morbidity, delayed diagnosis, pregnancy, and other aspects of the natural history of HoFH Know the person’s concept of what it means to be healthy and in control of care Know any internal family issues with regard to care and understanding of the implications of an FH diagnosis Understand any barriers to care as prescribed Include people with HoFH in decision making: integrate family history and experiences into discussions and know past medication history and any issues with adherence
Care Coordination	Identify a specific resource for families to contact with regard to care coordination and financial and social support Utilize resources to make adherence to treatment easier including pharmacists, genetic counselors, and nutrition experts Recognize the need for psychosocial support Facilitate consultation at key life course moments (e.g., cardiology care for medical events, obstetrics for pregnancy, lipid specialists for family members in different geographic regions)
Social Determinants of Health	Know the family’s financial and personal resources and how these might impact care Explore cultural beliefs in relation to care Understand any barriers to care created by personal circumstance

**Table 6.** Multidisciplinary Team Approach

Strategy	Considerations
Multidisciplinary care team	Expertise includes cardiologist, lipidologist, genetics professional.
Person with HoFH as team member	Promote shared decision making with the team and share information and tools. Empower the person with HoFH to voice their values.
Flexibility	Promote adaptability to serve the needs of each person with HoFH. The team composition may change based on the person’s needs (core versus extended team members as needed).
Community of Practice	Groups can learn together generating tacit and explicit knowledge-sharing.^50–53^
